# Evaluation of the effectiveness of a nationwide precision medicine program for patients with advanced non-small cell lung cancer in Germany: a historical cohort analysis

**DOI:** 10.1016/j.lanepe.2023.100788

**Published:** 2023-11-22

**Authors:** Anika Kästner, Anna Kron, Neeltje van den Berg, Kilson Moon, Matthias Scheffler, Gerhard Schillinger, Natalie Pelusi, Nils Hartmann, Damian Tobias Rieke, Susann Stephan-Falkenau, Martin Schuler, Martin Wermke, Wilko Weichert, Frederick Klauschen, Florian Haller, Horst-Dieter Hummel, Martin Sebastian, Stefan Gattenlöhner, Carsten Bokemeyer, Irene Esposito, Florian Jakobs, Christof von Kalle, Reinhard Büttner, Jürgen Wolf, Wolfgang Hoffmann

**Affiliations:** a Institute for Community Medicine, Section Epidemiology of Health Care and Community Health, University Medicine Greifswald, Greifswald, Germany; b National Network Genomic Medicine Lung Cancer, Germany; c Department I of Internal Medicine, Center for Integrated Oncology Aachen Bonn Cologne Duesseldorf, Lung Cancer Group Cologne, University Hospital of Cologne, Cologne, Germany; d Federal Association of the AOK, Berlin, Germany; e Institute of Pathology, University Hospital Bonn, Bonn, Germany; f Institute of Pathology, University Medical Center, Johannes Gutenberg University Mainz, Mainz, Germany; g Charité Comprehensive Cancer Center, Charité Universitätsmedizin Berlin, Berlin, Germany; h Institute of Pathology, Helios Klinikum Emil von Behring, Berlin, Germany; i West German Cancer Center, University Hospital Essen, Essen, Germany; j Clinic for Internal Medicine I, University Hospital Carl Gustav Carus and Medical Faculty of the TU Dresden, Dresden, Germany; k Institute of Pathology, Technical University of Munich (TUM), Munich, Germany; l Institute of Pathology, Ludwig-Maximilians-University (LMU), Munich, Germany; m Institute of Pathology, Friedrich-Alexander University Erlangen-Nuremberg, University Hospital Erlangen, Erlangen, Germany; n Translational Oncology/Early Clinical Trial Unit (ECTU), Comprehensive Cancer Center Mainfranken and Bavarian Cancer Research Center (BZKF), University Hospital Würzburg, Würzburg, Germany; o Department of Medicine II, Hematology/Oncology, University Hospital Frankfurt, Frankfurt, Germany; p Department of Pathology, University Hospital Giessen and Marburg, Giessen, Germany; q University Cancer Center Hamburg, University Medical Center Hamburg-Eppendorf, Hamburg, Germany; r Institute of Pathology, Heinrich-Heine-University and University Hospital Duesseldorf, Duesseldorf, Germany; s Department of Hematology and Stem Cell Transplantation, Faculty of Medicine and University Hospital Essen, University of Duisburg-Essen, Essen, Germany; t Berlin Institute of Health at Charité Universitätsmedizin Berlin, Berlin, Germany; u Institute of Pathology, Faculty of Medicine and University Hospital Cologne, Center for Integrated Oncology Aachen Bonn Cologne Duesseldorf, Lung Cancer Group Cologne, University of Cologne, Cologne, Germany

**Keywords:** Non-small cell lung cancer, Personalized medicine, Precision medicine program, Targeted therapies, Real-world data

## Abstract

**Background:**

The national Network Genomic Medicine (nNGM) Lung Cancer provides comprehensive and high-quality multiplex molecular diagnostics and standardized personalized treatment recommendation for patients with advanced non-small cell lung cancer (aNSCLC) in Germany. The primary aim of this study was to investigate the effectiveness of the nNGM precision medicine program in terms of overall survival (OS) using real-world data (RWD).

**Methods:**

A historical nationwide cohort analysis of patients with aNSCLC and initial diagnosis between 04/2019 and 06/2020 was conducted to compare treatment and OS of patients with and without nNGM-participation. Patients participating within the nNGM (nNGM group) were selected based on a prospective nNGM database. The electronic health records (EHR) of the prospective nNGM database were case-specifically linked to claims data (AOK, German health insurance). The control group was selected from claims data of patients receiving usual care without nNGM-participation (non-nNGM group). The minimum follow-up period was six months.

**Findings:**

Overall, n = 509 patients in the nNGM group and n = 7213 patients in the non-nNGM group met the inclusion criteria. Patients participating in the nNGM had a significantly improved OS compared to the non-nNGM group (median OS: 10.5 months vs. 8.7 months, *p* = 0.008, HR = 0.84, 95% CI: 0.74–0.95). The 1-year survival rates were 46.8% (nNGM) and 41.3% (non-nNGM). The use of approved tyrosine kinase inhibitors (TKI) in the first-line setting was significantly higher in the nNGM group than in the non-nNGM group (nNGM: 8.4% (43/509) vs. non-nNGM: 5.1% (366/7213), *p* = 0.001). Overall, patients receiving first-line TKI treatment had significantly higher 1-year OS rates than patients treated with PD-1/PD-L1 inhibitors and/or chemotherapy (67.2% vs. 40.2%, *p* < 0.001).

**Interpretation:**

This is the first study to demonstrate a significant survival benefit and higher utilization of targeted therapies for aNSCLC patients participating within nNGM. Our data indicate that precision medicine programs can enhance collaborative personalized lung cancer care and promote the implementation of treatment innovations and the latest scientific knowledge into clinical routine care.

**Funding:**

The study was funded by the AOK Federal Association Germany.

**Research in context**

**Evidence before this study**

Over the last decades, systemic therapies for patients with advanced non-small cell lung cancer (NSCLC) have rapidly evolved. Previous studies have shown that personalized therapies, which comprise the use of targeted therapies based on the detection of so-called driver mutations in tumor tissue or blood, substantially prolong survival compared to chemotherapy. However, access for patients outside specialized centers to these innovations remains a challenge. To evaluate the effectiveness of precision medicine programs for patients with advanced NSCLC in terms of survival, a PubMed literature search was conducted using the following search terms: (“non-small cell lung cancer” OR “NSCLC”) AND (“advanced” OR “metastatic”) AND (“precision medicine” OR “personalized medicine” OR “molecular profiling” OR “biomarker testing” OR “genomic sequencing”) AND (“usual care” OR “standard care” OR “routine care” OR “real-world” OR “community”) AND (“overall survival” OR “survival”). One study found that broad-based molecular diagnostics compared to routine mutation testing has no survival benefit, however, we found no study examining the effectiveness of a precision medicine program in terms of survival in the real-world setting.

**Added value of this study**

This is the first study to evaluate the effectiveness of a structured precision medicine program in patients with initial diagnosis of advanced NSCLC. The German national Network Genomic Medicine (nNGM) lung cancer performs standardized molecular diagnostics based on next generation sequencing (NGS), which comprises all known therapeutically relevant driver mutations and provides standardized personalized treatment recommendations to practitioners based on the molecular test result. Patients with advanced NSCLC participating in the nNGM were compared to a control group treated by practitioners in routine care without nNGM participation. The control group was based on claims data from a German statutory health insurance. We found a higher use of targeted therapies in the first-line setting in patients with nNGM participation, which was associated with a significantly prolonged survival.

**Implications of all the available evidence**

The complexity of precision medicine will continue to increase in the coming years due to the discovery of new driver mutations, development of new drugs and increasing drug resistance to targeted therapies. Practitioners in routine care are confronted with increasingly complex treatment algorithms that not only demand administrative and time resources, but also pose challenges for transferring the latest scientific knowledge from research to practice. Precision medicine programs, such as the nNGM, provide practitioners with standardized molecular diagnostics and structured treatment information, thus facilitating the implementation of precision medicine in the real-world care settings and thereby improving patient outcomes.

## Introduction

Overall, lung cancer is the second most frequently diagnosed cancer and the leading cause of cancer death in 2020 worldwide, representing 11.4% of cancers diagnosed and 18.0% of cancer deaths.^1^ In Germany, more than 50% of all patients with lung cancer are diagnosed at stage IV, with a relative 5-year survival rate of only 4–7%.^2^

Over the past decades, groundbreaking progresses have been made in understanding the pathogenesis of non-small cell lung cancer (NSCLC) and developing anticancer therapies based thereon.^3^ The era of personalized medicine in advanced NSCLC (aNSCLC) was initiated in 2004 by the landmark discovery of epidermal growth factor receptor (*EGFR*) mutations in patients that responded to the tyrosine kinase inhibitor (TKI) gefitinib, shifting the “*one size fits all*” approach to personalized treatment choices based on molecular characteristics of the tumor, simultaneously considering individual patient characteristics.^4^, ^5^, ^6^ Since then, an impressively prolonged overall survival (OS) benefit of mutation-directed therapies compared to chemotherapy has been reported for patients with aNSCLC in real-world data (RWD) as well as in clinical trials.^7^, ^8^, ^9^, ^10^, ^11^, ^12^, ^13^ To date, the European Medicines Agency (EMA) and US Food and Drug Administration (FDA) have approved targeted therapies to treat *EGFR* mutations, *ALK* and *ROS-1* fusions, *BRAF V600* mutations, *RET* fusions, *MET* exon 14 skipping mutations, *NTRK* 1–3 fusions and *KRAS G12C* mutations in the first- or later-line settings in patients with aNSCLC.^14^^,^^15^ Thus, the identification of driver mutations, for which approved targeted therapies are available, has opened promising treatment approaches for these patients, and further case-targeted therapies are under investigation.^7^, ^8^, ^9^, ^10^^,^^16^ In Germany, targetable genetic alterations are found in 30% of patients with aNSCLC.^17^

The European Society for Medical Oncology (ESMO) recommends that molecular testing for therapeutically relevant driver mutations should be systematically performed using next-generation sequencing (NGS)-based techniques in aNSCLC.^16^^,^^18^ However, recent data from a German registry (CRISP) showed that the overall testing rates for *EGFR*, *ALK*, *ROS1*, and *BRAF* in non-squamous aNSCLC were 72.5%, 74.5%, 66.1%, and 53.0%, respectively.^19^ The percentage of patients with testing for all known therapeutically relevant mutations was strikingly lower.^19^ Different reasons underlie these observations. In clinical practice, oncologists treat a variety of different tumor entities, and due to the dynamic emergence of oncogenic driver alterations, the development of targeted drugs, and the frequent initiation of clinical trials in lung cancer, the implementation of personalized cancer care for aNSCLC in routine care is challenging and pertinent guidelines may not reflect the latest knowledge.^19^, ^20^, ^21^ Due to the increasing complexity of available molecular data, it is essential to support clinicians in their therapeutic decisions.^22^

Therefore, novel implementation strategies of precision medicine are needed to ensure high-quality evidence-based routine care for patients with aNSCLC. One promising strategy is to establish national precision medicine programs that provide broad molecular diagnostics based on NGS, clinical decision support and clinical trial matching outside the academic setting.^23^ This is also supported by ESMO, stating that the use of off-label drugs matched to genomics should only be done if an access program and a procedure of decision making has been developed at the regional or national level. In addition, it is recommended that clinical research centers use multigene sequencing as a tool to screen patients eligible for clinical trials, to accelerate drug development, and to prospectively collect data that could provide further information to optimize the use of this technology.^18^

The Network Genomic Medicine (NGM) Lung Cancer was established in Germany at the Center for Integrated Oncology (CIO) of the University Hospital of Cologne in 2010 and expanded in 2018 to the national Network Genomic Medicine Lung Cancer (nNGM) with funding from the German Cancer Aid (DKH) to provide high-quality molecular diagnostics for patients with aNSCLC, to rapidly develop and initiate clinical trials for new individualized treatment approaches, and to provide counseling and personalized treatment decision support to clinical oncologists and patients nationwide.^24^^,^^25^ Numerous German statutory health insurance companies, such as the AOK, support the nNGM by covering the costs of the NGS-based molecular diagnostics and counseling on the basis of special care contracts (SCC) according to §140a of the Fifth Book of the German Social Code (SGB V). The AOK is one of the largest statutory health insurance providers in Germany, with a market share of approximately one-third of the total population. In total, cost coverage for nNGM is granted for about 93% of statutory insured lung cancer patients in Germany.

To date, several studies have investigated the impact of broad genomic sequencing compared to no testing or limited testing (*EGFR* and/or *ALK* only), the impact of a biomarker-based (personalized) cancer treatment strategy and the feasibility of a nationwide molecular profiling program.^8^, ^9^, ^10^^,^^26^, ^27^, ^28^, ^29^ However, to our knowledge, no study has compared the impact of such a large-scale community-based precision medicine program for aNSCLC on the patients’ outcome compared to routine clinical care.^30^ This is the first study evaluating the effectiveness of the nNGM in terms of overall survival (OS) in patients with aNSCLC using RWD.

## Methods

Patients with unresectable and advanced NSCLC with initial diagnosis between the 1st of April 2019 and the 30th of June 2020, who were insured by the German statutory health insurance AOK, were included in this historical cohort study. Patients who received NGS-based molecular diagnostics and treatment decision support within nNGM (nNGM group) were compared with patients who received clinical routine care without nNGM-participation (non-nNGM group). For this purpose, the nNGM electronic health records (EHR) of the prospective nNGM database of the nNGM group were case-specifically linked with AOK health insurance data. The non-nNGM group consisted of aNSCLC patients who were also insured by the AOK, but were not treated within the nNGM. A brief description of the nNGM is provided in the Supplementary Material.

### Electronic health records and molecular diagnostics of the nNGM

The electronic health records (EHRs) of the nNGM patients contained baseline data such as patient characteristics, date of initial diagnosis, tumor stage and results of multiplex molecular analysis (including driver mutations and mutation status). In the nNGM centers, harmonized molecular testing is conducted for all patients with initial diagnosis of aNSCLC. Briefly, nNGM uses a multiplex testing approach combined with deep sequencing to detect rare gene mutations. This strategy involves multiple techniques, such as NGS based on deoxyribonucleic acid (DNA) and ribonucleic acid (RNA), immunohistochemical detection (IHC) of protein expression (such as Programmed cell death ligand-1 (PD-L1)) and fluorescence in situ hybridization (FISH). The number of genes to be tested as well as the methods used are updated regularly based on latest knowledge. During the study period, the nNGM-lung cancer panel for DNA-based NGS included 21 genes to detect mutations, among others, in *EGFR*, *KRAS*, *BRAF*, *PIK3CA*, *HER2*, *TP53* and *MET*. Fusions in *ALK*, *RET*, *ROS1*, *HER2, NTRK1-3* and *FGFR1-3* were detected by FISH and/or RNA-based NGS, and amplifications in *HER2*, *MET* and *FGFR1* usually by FISH. *MET* exon 14 skipping mutations were detected by DNA- and RNA-based NGS. All testing was performed using already established and published techniques. In particular, DNA- and RNA-based NGS has been conducted as described in Riedel et al. (in the Supplementary Data),^31^ FISH analyses were conducted according to Heydt et al.,^32^ and PD-L1 IHC was performed as described in Scheel et al.^33^ The aforementioned baseline and molecular data were prospectively collected in a central nNGM database. The provided nNGM-EHRs did not contain information on further inpatient and outpatient treatments. Therefore, to examine the treatment course and outcomes of nNGM patients, the EHRs were case-specifically linked to administrative claims data based on pseudonyms by an independent trusted third party.^34^

### Claims data

In the current study, administrative inpatient and outpatient claims data from the German statutory health insurance AOK were provided by the Research Institute of the Local Health Care Funds (WIdO). The dataset for this study included general demographic data (month of birth, sex assigned at birth, federal state of residence, and date of death) and information on outpatient treatment (treatment dates, procedures, diagnosis codes and drug therapy) as well as inpatient treatment (treatment dates, diagnoses codes, procedures, and length of hospital stay). Inpatient and outpatient diagnosis data were coded according to the International Classification of Diseases, 10th revision, German Modification (ICD-10-GM).^35^ Outpatient drug therapies were classified using Anatomical Therapeutic Chemical (ATC) codes.^36^ Furthermore, outpatient and inpatient therapies were classified based on operation and procedure codes (OPS).^37^ With the start of reimbursement based on the special care contract, the AOK data covered a period from 1st of April 2019 to 31st of December 2020, ensuring a minimum follow-up since initial diagnosis of six months and a maximum follow-up of 21 months for all aNSCLC cases. Ethical approval was not required as the claims data did not contain personally identifiable information.

### Tyrosine kinase inhibitors (TKI) and PD-1/PD-1 inhibitors

The following TKI treatments that were approved during the study period were examined in the analysis: erlotinib, gefitinib, afatinib, dacomitinib, osimertinib, crizotinib, alectinib, brigatinib, ceritinib, lorlatinib, and entrectinib. Furthermore, the serine/threonine kinase inhibitors dabrafenib and trametinib were included and categorized as TKIs in the following. Other off-label TKI treatments considered in the analysis were cabozantinib, sorafenib, and vemurafenib. The PD-1/PD-L1 inhibitors (immunotherapy) considered in this analysis were pembrolizumab, atezolizumab, nivolumab, durvalumab, and cemiplimab.

### Patient selection

All patients aged ≥18 years with an initial diagnosis of aNSCLC between 1st of April 2019 and 30th of June 2020 in Germany, who were insured with the AOK and received systemic lung cancer related therapy were eligible for this analysis.

Patients with incident aNSCLC who received systemic lung cancer related therapy were selected based on claims data. Therefore, various inclusion and exclusion criteria were defined for both groups. The detailed description of the selection criteria is presented in Supplementary Table S1, and the flow chart for patient selection is shown in Supplementary Fig. S1. To detect aNSCLC patients, proxy variables were used to exclude patients with lower tumor stages with tumor resection and/or radiotherapy, and patients with SCLC. Therefore, patients with no main diagnosis lung cancer and lung cancer related treatment (according to the current German S3 guideline lung cancer^15^) within the first six months after initial diagnosis were excluded (to exclude non-advanced incident NSCLC patients), as well as patients with lung resection (to exclude patients with resectable NSCLC), combination therapy with platinum-containing drugs in combination with etoposide (to exclude patients with SCLC), and patients receiving radiotherapy without metastasis coding (to exclude patients with tumor stage IIIA).

### Statistical analysis

Categorical variables are presented as absolute numbers and percentages, and continuous data as means and 95% confidence intervals (95% CI). A *t*-test was used to compare continuous data, and a chi-square test was conducted to compare categorical variables.

The first-line (1L) and second-line (2L) treatments within the first six months since initiation of 1L therapy were analyzed, as due to the minimum follow-up period information on the course of treatment was largely available for both groups. Treatment was categorized as TKI, mono-immunotherapy (mono-IO), immuno-chemotherapy (IO-CT) or chemotherapy (CT) as follows: Patients who received mono- or polychemotherapy without the use of TKIs or immunotherapies were grouped into the chemotherapy group. If at least one TKI was administered, patients were grouped into the TKI group. Patients who received PD-1/PD-L1 inhibitors with or without chemotherapy were grouped into the mono-immunotherapy (mono-IO) or immuno-chemotherapy (IO-CT) group.

Overall survival (OS) was defined as the time from initiation of 1L therapy to death or last follow-up. Patients who were still alive at the end of the study period were censored. Kaplan–Meier analysis was used to compare the OS of the nNGM group with that of the non-nNGM group overall, and differentiated by 1L treatment group and treatment within the first six months after start of 1L treatment. The log-rank test was applied to analyze the differences between the survival curves. The hazard ratio (HR) was determined by multivariable Cox regression analysis adjusted for age and gender and is reported with its 95% confidence interval (95% CI). The Cox proportional hazard assumption was assessed with the supremum test. Furthermore, maturity of the survival data was assumed, if more than 50% of events were observed during the study period. The follow-up period was defined as time from initiation of 1L therapy until last follow-up.

All statistical analyses were performed using SAS Software release 9.4 (Version 9.4; SAS Institute, Cary, NC, USA) and Python version 3.8. All statistical analyses were considered to be statistically significant if the two-sided *p*-value was less than 0.05.

### Role of the funding source

This study was funded by the AOK Federal Association Germany. The funder had no role in the study design, data analysis, or preparation of the manuscript.

## Results

### Patient selection and baseline characteristics

Patients with aNSCLC with initial diagnosis between 1st of April 2019 and 30th of June 2020 in Germany, who were insured with the AOK and received systemic lung cancer related therapy were included in the analysis. In total, data from 631 nNGM patients and AOK claims data for 12914 non-nNGM patients were available. After applying the inclusion and exclusion criteria, the nNGM group comprised 509 patients and the non-nNGM group 7213 patients (see Supplementary Fig. S1). The patients of the nNGM group had a mean age of 67.6 and the patients of the non-nNGM group of 67.5 years (*p* = 0.971), respectively, with 37.9% and 38.6% female patients (*p* = 0.985, see Table 1). No missing values were present in any of the reported variables. All patients in the nNGM group received the molecular diagnostics as described in the methods section.

**Table 1:** Patient characteristics and systemic therapies received within the first six months after initiation of first-line treatment.

	nNGM group (n = 509)		non-nNGM group (n = 7213)		*p*-value
**Age, mean (95% CI)**	67.6 (66.7, 68.4)		67.5 (67.3, 67.8)		0.971
**Female, n (%)**	193	37.9%	2783	38.6%	0.985
**1L treatment, n (%)**					
Total	509	100.0%	7213	100.0%	
TKI	43	8.4%	366	5.1%	0.001
Mono-IO	62	12.2%	768	10.6%	0.280
IO-CT	180	35.4%	2399	33.3%	0.331
CT	224	44.0%	3680	51.0%	0.002
**2L treatment, n (%)**					
Total	145	28.5%	1692	23.5%	0.010
TKI	14	9.7%	96	5.7%	0.053
Mono-IO	46	31.7%	557	32.9%	0.769
IO-CT	31	21.4%	233	13.8%	0.012
CT	54	37.2%	806	47.6%	0.016

nNGM, national Network Genomic Medicine Lung Cancer; 1L, first line; 2L, second line; TKI, tyrosine kinase inhibitor; mono-IO, mono-immunotherapy; IO-CT, immuno-chemotherapy; CT, chemotherapy.

### Overall survival

**Fig. 1:**
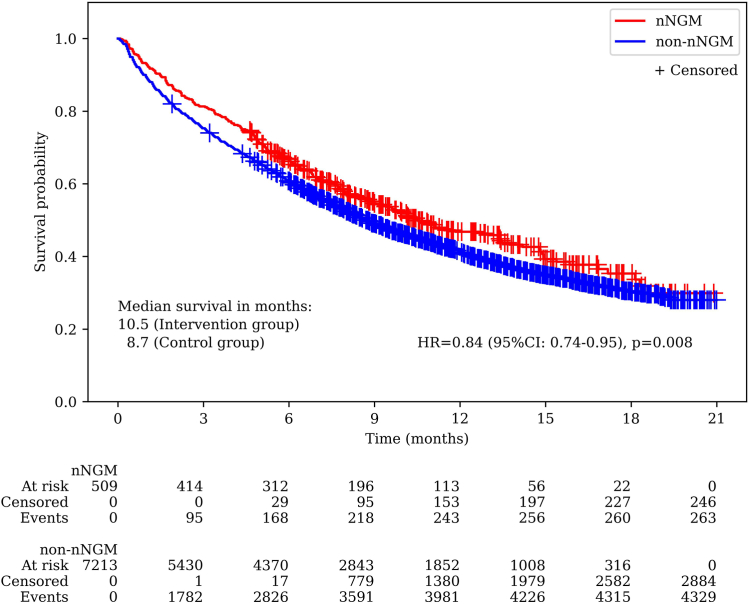
Kaplan–Meier survival curve comparing overall survival since initiation of first-line treatment between nNGM group and non-nNGM group.

**Fig. 2:**
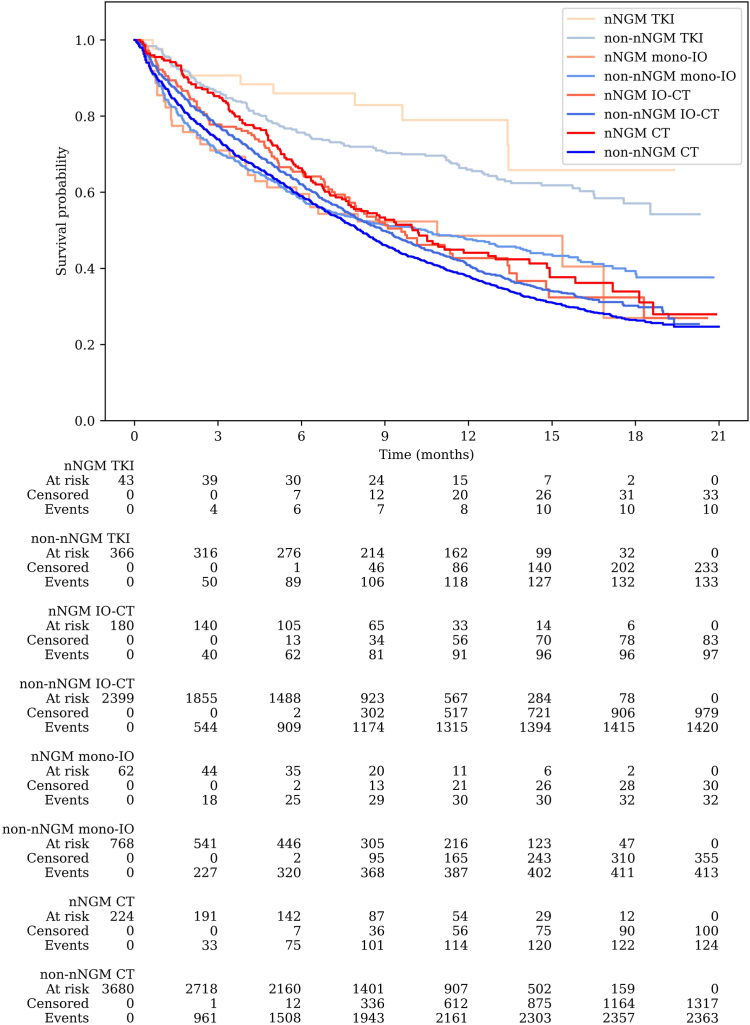
Kaplan–Meier survival curve comparing overall survival of the nNGM group and non-nNGM group stratified by first-line treatment (TKI vs. mono-immunotherapy [mono-IO] vs. immuno-chemotherapy [IO-CT] vs. chemotherapy [CT]).

The median follow-up time since start of 1L treatment was 7.5 months both in the nNGM and non-nNGM group. The mature OS data (59.5% of events) showed significantly prolonged OS in patients who received molecular diagnostics and treatment information in the nNGM compared to patients in routine care, with a median survival time of 10.5 months for the nNGM group and 8.7 months for the non-nNGM group (adjusted HR = 0.837, 95% CI: 0.74–0.95, *p* = 0.008; Fig. 1). The OS rate for the nNGM group was 66.8% at six months and 46.8% at one year, while the OS rate for the non-nNGM group was 60.8% at six months and 41.3% at one year. Furthermore, Fig. 2 shows the OS for the nNGM and non-nNGM group categorized by the type of 1L therapy. The 1-year OS rates in the nNGM group were 79.0% with TKI treatment, 48.6% with mono-IO, 42.7% with IO-CT and 44.1% with CT, whereas in the non-nNGM group the 1-year OS rates were 66.0% (*p* = 0.204), 47.6% (*p* = 0.929), 40.9% (*p* = 0.549) and 37.9% (*p* = 0.015). Overall, patients receiving a TKI as 1L treatment had a significantly higher 1-year OS rate compared to patients treated with immunotherapy and/or chemotherapy in the 1L setting (TKI: 67.2% vs. immunotherapy and/or chemotherapy: 40.2%; *p* < 0.001). Supplementary Fig. S2 additionally shows the comparison of OS between the nNGM group and the non-nNGM group further grouped by the treatment within the first 6 months after the start of 1L treatment (TKI therapy at least once within the first 6 months after the start of 1L treatment vs. IO at least once within the first 6 months vs. neither TKI nor IO within the first 6 months).

### Therapy within six months after initiation of first-line treatment

The proportion of patients in the nNGM group receiving TKI treatment as their 1L treatment was significantly higher compared to the non-nNGM group (8.4% vs. 5.1%, *p* = 0.001, Table 1). Overall, 28.5% of the nNGM patients received 2L treatment within the first six months after treatment initiation compared to 23.5% of patients in non-nNGM group (*p* = 0.010). In the second therapy line, the nNGM group had a trend towards a higher proportion of patients receiving TKI treatment (9.7% vs. 5.7%, *p* = 0.053) compared to the non-nNGM group. Overall, a significantly lower proportion of patients in the nNGM group received chemotherapy as their 1L and 2L treatment compared to the non-nNGM group (1L: 44.0% vs. 51.0%, *p* = 0.002; 2L: 37.2% vs. 47.6%, *p* = 0.016). The usage of mono-immunotherapies both in the 1L and 2L was comparable between the groups (1L: 12.2% in nNGM vs. 10.6% in non-nNGM group, *p* = 0.280; 2L: 31.7% in nNGM vs. 32.9% in non-nNGM group, *p* = 0.769). The proportion of patients receiving immuno-chemotherapies was comparable in the 1L (nNGM: 35.4% vs. non-nNGM: 33.3%, *p* = 0.331), however, a significantly higher proportion received immuno-chemotherapies in the 2L in the nNGM group compared to the non-nNGM group (21.4% vs. 13.8%, *p* = 0.012). A detailed overview of the TKIs and PD-1/PD-L1 inhibitors administered as 1L and 2L therapy within the first six months after treatment initiation is provided in Table 2. None of the nNGM patients and n = 13 (0.2%) of the non-nNGM patients received off-label TKI treatments within the first six months after treatment initiation.

**Table 2:** Detailed description of TKIs and PD-1/PD-L1 inhibitors administered in the first-line (1L) and second-line (2L) setting within the first six months after initiation of first-line treatment (the percentages of 1L and 2L therapies both refer to the total number of patients per group).

	nNGM group (n = 509)						non-nNGM group (n = 7213)					
	1L		2L		Total		1L		2L		Total	
	n	%	n	%	n	%	n	%	n	%	n	%
**TKI**	43	8.4%	14	2.8%	57	11.2%	366	5.1%	96	1.3%	462	6.4%
Afatinib	3	0.6%	–	–	3	0.6%	31	0.4%	15	0.2%	46	0.6%
Alectinib	8	1.6%	1	0.2%	9	1.8%	55	0.8%	13	0.2%	68	0.9%
Brigatinib	–	–	1	0.2%	1	0.2%	–	–	–	–	–	–
Cabozantinib	–	–	–	–	–	–	7	0.1%	2	<0.1%	9	0.1%
Ceritinib	–	–	2	0.4%	2	0.4%	1	<0.1%	–	–	1	<0.1%
Crizotinib	6	1.2%	1	0.2%	7	1.4%	25	0.3%	11	0.2%	36	0.5%
Dabrafenib + Trametinib	5	1.0%	1	0.2%	6	1.2%	33	0.5%	8	0.1%	41	0.6%
Dacomitinib	–	–	–	–	–	–	5	0.1%	–	–	5	0.1%
Entrectinib	–	–	–	–	–	–	–	–	–	–	–	–
Erlotinib	1	0.2%	2	0.4%	3	0.6%	5	0.1%	3	<0.1%	8	0.1%
Gefitinib	1	0.2%	–	–	1	0.2%	4	0.1%	1	<0.1%	5	0.1%
Lorlatinib	–	–	–	–	–	–	–	–	1	<0.1%	1	<0.1%
Osimertinib	19	3.7%	6	1.2%	25	4.9%	198	2.7%	42	0.6%	240	3.3%
Sorafenib	–	–	–	–	–	–	2	<0.1%	–	–	2	<0.1%
Vemurafenib	–	–	–	–	–	–	1	<0.1%	1	<0.1%	2	<0.1%
**PD-1/PD-L1 inhibitor**	242	47.5%	77	15.1%	319	62.7%	3167	43.9%	790	11.0%	3957	54.9%
Pembrolizumab	231	45.4%	40	7.9%	271	53.2%	2569	35.6%	323	4.5%	2892	40.1%
Atezolizumab	11	2.2%	8	1.6%	19	3.7%	535	7.4%	215	3.0%	750	10.4%
Nivolumab	–	–	13	2.6%	13	2.6%	60	0.8%	79	1.1%	139	1.9%
Durvalumab	–	–	18	3.5%	18	3.5%	4	0.1%	184	2.6%	188	2.6%
Cemiplimab	–	–	–	–	–	–	4	0.1%	–	–	4	0.1%

nNGM, national Network Genomic Medicine Lung Cancer; 1L, first line; 2L, second line; TKI, tyrosine kinase inhibitor; PD-1, programmed cell death 1; PD-L1, programmed cell death ligand-1.

**Note**: The minus sign indicates that no medication was administered (n = 0).

## Discussion

In this study, for the first time, the survival benefits of a precision medicine program for aNSCLC providing broad NGS-based molecular diagnostics and treatment decision support for clinicians including recommendations for off-label therapies and clinical trials in the community-based setting are demonstrated using RWD. We found that a significantly higher proportion of patients, who participated within the nNGM received targeted therapies and less frequently chemotherapy, which was associated with a significantly improved overall survival.

As precision medicine moves from academic settings into community practice, oncologists face clinical, financial, and administrative challenges in implementing these treatments.^23^ These challenges include determining which molecular tests are best to use and when tissue should be tested, interpreting the test results and determining actionability, understanding the role of genetic counseling and/or follow-up testing, determining clinical trial eligibility, and assessing patient attitudes and financial concerns.^38^ Given the multifaceted, complex and rapidly changing landscape in precision medicine, it is imperative to establish early and frequent communication among all stakeholders.^38^ One key component for the effective implementation of precision medicine in oncology practice is the data infrastructure for decision support, such as standardized testing algorithms and treatment guidance for physicians.^39^ The aforementioned aspects have been implemented within the framework of the nNGM with the primary goal of providing harmonized, standardized, comprehensive and high-quality molecular diagnostics of tumor tissue for every lung cancer patient in Germany.^40^

Previous studies using RWD have highlighted the benefits of personalized care, in particular broad molecular testing, for patients with aNSCLC and demonstrated improved OS in patients with driver-mutation positive tumors.^8^, ^9^, ^10^ However, these studies compared patients within defined molecular screening programs with presence of oncogenic driver mutation and genotype-directed therapy with patients without any oncogenic drivers, but did not address the impact of implementing a precision medicine program into a national health system. Bruno et al. investigated the effectiveness of implementing a precision medicine thoracic service in a large academic-community practice network and found significantly higher rates of NGS testing for patients with stage IV NSCLC and a trend towards higher rates of actionable alterations.^41^ However, the control group consisted of patients treated prior to the implementation of the program, potentially introducing a chronology bias, and clinical outcomes such as OS were not studied. Presley et al. investigated the impact of broad genomic sequencing vs. routine testing (*EGFR* and/or *ALK* only) in patients with aNSCLC and found comparable survival rates between the groups.^26^ However, only patients with documentation of either broad genomic sequencing testing or specific *EGFR* mutation and/or *ALK* rearrangement testing (as standard-of-care) were considered.^26^ Thus, the effectiveness of a precision medicine program was not studied, especially since, as previously reported, in community-based clinical routine care, not all patients with aNSCLC receive molecular diagnostics, and the study population did not represent usual care of the general aNSCLC population and clinicians, e.g., did not receive treatment support. Overall, the impact of the personalized treatment with the implementation of molecular diagnostics and targeted therapy in patients with aNSCLC has been highlighted in previous studies, but the effectiveness of a precision medicine program remains unclear.

The importance of the use of RWD to study the effects of precision medicine has been recognized.^42^ In comparison to patients participating in randomized controlled trials, RWD more accurately reflect cancer patients in usual care, which are often older, more likely to be female, have a poorer performance status along with a worse disease prognosis, and are increasingly used to reevaluate drug efficacy.^43^, ^44^, ^45^, ^46^, ^47^ RWD for research purposes can be derived from a variety of data sources (e.g., administrative claims data, EHR or cancer registries), all of which have strengths and limitations. Claim data provide a reliable record of the use of inpatient and outpatient healthcare services longitudinally over time. In Germany in particular, such data are valuable since, due to the statutory health insurance system, the data largely represent the general population.

Based on RWD from the German cancer registries, our study population well reflects the lung cancer patients in Germany. In 2018, 61.7% of lung cancer patients in Germany were male and the median age at the time of initial diagnosis was 70 years in men and 69 years in women.^2^ This is also in line with Hardtstock et al., who studied the treatment and survival of 1741 aNSCLC patients in Germany incident in 2012–2015 based on claims data and reported a median age of 68 years and 70.1% of aNSCLC patients were male.^48^ Hardtstock et al. found that during the study period across all treatment lines, 70.3% of patients received a chemotherapy only, 21.2% received a TKI-based therapy and immunotherapy without a TKI was prescribed in 4.5% of the patients.^48^ As expected, we observed that the proportion of aNSCLC patients with incidence in 2019–2020 who received chemotherapy substantially decreased to 44–51% in the first-line setting and immunotherapy increased to 44–48%, while interestingly, TKI prescription remained at 5.1% in the non-nNGM group and increased to 8.4% in the nNGM-group only. The 1-year survival rate of 47.9% was comparatively high over the observation period in the study by Hardtstock et al., however, survival was calculated starting from the date of initial diagnosis.^48^ In our study, despite the significantly more frequent use of immunotherapies, the 12-month survival rate in the non-nNGM group was 41.3%. Another RWD-study from Italy investigated the treatment and outcomes of 1787 patients with aNSCLC with first diagnosis between November 2014 to November 2015 and found a median OS of 9.34 months and a 12-month survival rate of 40%, although 16.1% of patients received a first-line targeted therapy.^49^ One study by Lester et al. using RWD (2016–2018) from the United Kingdom found a median OS of 9.5 months in 1003 aNSCLC patients.^50^ In that study, 69.6% received chemotherapy, 17.8% mono-immunotherapy and 12.6% targeted therapy with TKI as first-line treatment.^50^ Overall, our study reflects the real-world care of aNSCLC patients in Germany, thus for the first time demonstrating the benefits of a community-based precision medicine program.

Furthermore, a survival benefit of patients receiving chemotherapy without immunotherapy or targeted therapy within the nNGM was found. It is important to note that during the study period the majority of nNGM partners strongly collaborated with academic or certified lung cancer centers. Therefore, the survival benefit of patients with chemotherapy within the nNGM in our study might be due higher expertise and better supportive care. However, over the last years, the network has grown steadily and aims to collaborate with all lung cancer practitioners in Germany. Thus, ensuring the provision of personalized care for all aNSCLC patients in Germany at their local residential area outside of academic centers is the primary aim of the nNGM.

When evaluating the effectiveness of a precision medicine program, in addition to clinical benefits, cost-effectiveness must also be considered, particularly since NGS-based testing strategies are more comprehensive and thus potentially more expensive than single-gene tests. However, within nNGM, costs for molecular diagnostics and clinical recommendation are fully covered by the German statutory health insurance companies as part of special care contracts, and currently, no cost-effectiveness or cost-utility methods, including quality-adjusted life-years (QALY), are used by stakeholders for reimbursement decision making. Recently, however, studies have shown that NGS is a cost-effective strategy compared to single-gen testing in patients with aNSCLC and can increase the proportion of patients receiving biomarker-driven therapies.^51^, ^52^, ^53^ Due to scarce resources, these approaches and discussions on willingness to pay thresholds will likely become more relevant in Germany in the future.

The importance of structured and standardized precision medicine programs for aNSCLC patients will continue to grow with the discovery of targetable driver mutations and further approval of targeted therapies. Also, novel emerging diagnostic methods, as e.g., liquid biopsy to detect TKI resistance, as it is implemented in nNGM, will continue to gain importance.^54^^,^^55^ The significance of such programs for the quality of life of aNSCLC patients is largely unknown so far, although a number of clinical studies have already found positive effects on quality of life with the use of targeted therapies compared to chemotherapy,^56^^,^^57^ indicating that positive effects may also be presumed in this regard. Other important concepts that the structure of precision medicine programs could facilitate in the future would be the expansion of molecular tumor boards at the time of initial diagnosis, which in Germany currently primarily provide treatment recommendations for patients beyond the first-line setting. With the increase in driver mutations and the knowledge on the relevance of co-occurring mutations in the first-line setting, it is a challenge for conventional guidelines to provide treatment recommendations based on latest knowledge and thus the provision of expert knowledge is essential.

One limitation of this study is that since claim data is generated for billing purposes, important clinical and laboratory health information was lacking, such as tumor histopathology, tumor stage, laboratory parameters, mutation status information, genomic biomarker testing results, and smoking status.^58^ Thus, proxy variables were used to identify patients with aNSCLC. Previously, several algorithms have been proposed to identify patients with aNSCLC based on claims data.^59^, ^60^, ^61^ To detect patients with incident aNSCLC, we first included patients who had a lung cancer diagnosis in combination with a lung cancer related systemic therapy according to the current German S3 lung cancer guideline within the first six months after the initial lung cancer diagnosis.^15^ Furthermore, to exclude patients with resectable and non-advanced NSCLC, patients with surgical therapy or patients who received radiotherapy without presence of metastases were excluded. Patients with SCLC were also excluded on the basis of proxy variables. Since SCLC patients in Germany receive platinum-based chemotherapy (cis- or carboplatin in combination with etoposide) plus optionally atezolizumab as standard first-line therapy,^62^^,^^63^ and due to the high reliability of the claims data, we are confident that we could reliably exclude those patients. Therefore, we only considered patients with systemic treatment in the analysis, as otherwise the entity definition would not have been sufficiently specific. The encoded procedures and accounted medications allow conclusions about the treatment courses, but are also prone to errors, e.g., due to participation in studies, that would result in missing invoices to the statutory insurances. However, based on the ICD-10 coding of metastases and diagnoses, we found that the proportion of patients with stage IV NSCLC (nNGM group: 86%, non-nNGM group: 88%) as well as the proportion of patients with secondary carcinomas (nNGM group: 1.2%, non-nNGM group: 2.3%) were comparable between the groups. Another limitation was the lacking assessment of progression-free survival and patient-reported outcomes, as those are not documented in claims data. Also, no power calculation was performed in advance when designing the study, however, based on the observed sample size and relevant parameters (i.e., the estimated hazard ratio and the survival probability of the non-nNGM group at 21 months at the end of follow-up), we determined a power for a log-rank test of 85%, indicating that our study was not overpowered. Furthermore, it remains unclear whether molecular diagnostics were performed in the control group, and if so, which biomarkers were examined and which alterations were detected, thus the proportion of patients with personalized care cannot be assessed. In addition, for data protection reasons, the exact location of the practitioners was not included in the available claims data. The effectiveness of the nNGM precision medicine program in terms of OS and quality of life for aNSCLC patients will be further investigated in the ongoing intervention study *DigiNet* (NCT05818449).

In conclusion, we herewith report the survival benefits of a nationwide precision medicine program for aNSCLC patients in a historical cohort analysis based on RWD. Patients receiving broad NGS-based molecular diagnostics and treatment decision support had significantly higher overall survival rates compared to patients receiving routine clinical care based on clinician’s choice. Precision medicine programs will continue to grow in importance in the future due to increasingly complex personalized therapy regimens, increasing TKI resistance and the growing number of multiple gene alterations. The successful implementation of the German national Network Genomic Medicine lung cancer further demonstrates high acceptance among practitioners and provides a model for a successful community-based structured program for personalized care of aNSCLC patients.

## Contributors

Conceptualization and methodology: AKä, AKr, nvdB, JW and WH. Data analysis plan: AKä, MSche and KM. Data collection and provision of nNGM data: AKr, MSche, NP, NH, DTR, SSF, MSchu, MW, WW, FK, FH, HDH, MSe, SG, CB, IE, FJ, CvK, RB and JW. Data provision of claims data: GS. Formal data analysis: KM. Writing—original draft preparation: AKä, AKr and nvdB. Writing—statistical analysis report: KM. Writing—review and editing: KM, MSche, JW and WH. Funding acquisition: nvdB and WH. AKä and KM directly accessed the data reported in the manuscript and take responsibility for the accuracy of the data analysis. All authors have contributed to the manuscript and agreed with the decision to submit for publication.

## Data sharing statement

The datasets generated and analyzed as part of the current study will not be made publicly available due to data protection reasons and contractual regulations with the AOK Federal Association Germany. For further information please contact the corresponding author.

## Ethics statement

The prospective data collection of patients participating within the nNGM was approved by the ethics committee of the University Hospital of Cologne (18–358 for nNGM data collection). All procedures performed in this study were in accordance with the ethical standards of the institutional and/or national research committee and with the 1964 Helsinki declaration and its later amendments or comparable ethical standards. Informed consent was obtained from all individuals participating within the nNGM. Ethical approval was not required for the provision of the administrative inpatient and outpatient claims data from the German statutory health insurance AOK as the claims data did not contain personally identifiable information.

## Declaration of interests

AKä, nvdB, KM, and WH declare financial support from the Federal Association of the AOK, Berlin, Germany (e.g., for performing the analyses and writing the manuscript), as stated in the manuscript. MSche received institutional grants from Amgen, Bristol Myers Squibb, Dracen Pharmaceuticals Inc., Janssen, Novartis, Siemens Healthineers, personal consulting fees from Amgen, AstraZeneca, Boehringer Ingelheim, Bristol Myers Squibb, Janssen, Novartis, Pfizer, Roche, Sanofi-Aventis, Siemens Healthineers and Takeda, honoraria or payment for educational events from Amgen, AstraZeneca, Bristol Myers Squibb, Novartis, Pfizer, Sanofi-Aventis and Takeda; received support for attending meetings and/or travel from Boehringer Ingelheim, Janssen, Pfizer, AstraZeneca; and reports participation on a Data Safety Monitoring Board at Boehringer Ingelheim, a leadership or fiduciary role (unpaid) at the ESMO Lung Cancer Faculty, ESMO Climate Change Task Force and the EORTC Lung Cancer group. DTR received payment or honoraria from Roche (Bristol Myers Squibb), Bayer and Lilly for lectures, presentations, speakers’ bureaus, manuscript writing or educational events and support for attending meetings and/or travel from Bayer. MSchu received institutional grants from AstraZeneca and Bristol Myers Squibb, consulting fees from Amgen, AstraZeneca, Blueprint Medicines, Boehringer Ingelheim, Bristol Myers Squibb, GlaxoSmithKline, Janssen, Merck Serono, Novartis, Roche, Sanofi-Aventis and Takeda, payment or honoraria from Amgen, Boehringer Ingelheim, Bristol Myers Squibb, Janssen, Merck Sharp & Dohme, Novartis, Roche, Sanofi-Aventis for lectures, presentations, speakers’ bureaus, manuscript writing or educational events; received support for attending meetings and/or travel from Janssen, Bristol Myers Squibb, Roche, Boehringer Ingelheim, Novartis, Amgen; and participated in Data Safety Monitoring Boards or Advisory Boards at Amgen, Bristol-Myers Squibb, Novartis, Sanofi-Aventis, Janssen, Tacalyx, Abalos and GlaxoSmithKline. MW received institutional grants from Roche, personal consulting fees from Bristol Myers Squibb, Novartis, Lilly, Boehringer Ingelheim, Amgen, Immatics, Bayer and ImCheck Therapeutics, payment or honoraria for educational events from Lilly, Boehringer Ingelheim, SYNLAB, Janssen, Merck Serono, GWT-TUD GmbH, Amgen and Novartis; he received support for attending meetings and/or travel from Pfizer, Bristol Myers Squibb, AstraZeneca, Janssen, Amgen, GEMoaB, Sanofi-Aventis, Immatics, Merck Serono and Daiichi Sankyo and honoraria for participation in a Data Safety Monitoring Board or Advisory Board of ISA Pharmaceuticals. FK received payment or honoraria from Bristol-Myers Squibb, Merck Sharp & Dohme, Novartis, Roche, Lilly, Agilent, Bayer, Merck and Trillium for lectures, presentations, speakers’ bureaus, manuscript writing or educational events and is a board member at the National Pathologists’ Association Germany; and received personal honoraria for lectures by AstraZeneca and Novartis and participated in an advisory board by AstraZeneca. MSe received grants from AstraZeneca and consulting fees from AstraZeneca, Bristol Myers Squibb, Merck Sharp & Dohme, Novartis, Lilly, Roche, Boehringer Ingelheim, Amgen, Takeda, Johnson, Merck-Serono and GSK; received payment or honoraria from AstraZeneca, Bristol Myers Squibb, Merck Sharp & Dohme, Novartis, Lilly, Roche, Boehringer Ingelheim, Amgen, Takeda, Johnson, CureVac, BioNTech, Merck-Serono, GSK, Daiichi and Pfizer for lectures, presentations, speakers’ bureaus, manuscript writing or educational events; and received support for attending meetings and/or travel from Pfizer, Bristol-Myers Squibb and participated in a Data Safety Monitoring Board at Amgen. CB received consulting fees from the AOK NRW-Hamburg, and honoraria for participation in a Data Safety Monitoring Board or Advisory Board of AstraZeneca, Bayer Healthcare, Bristol Myers Squibb, Janssen-Cilag, Merck Serono and Sanofi-Aventis; received payment or honoraria for lectures given for Bristol Myers Squibb, Roche Pharma and Sanofi-Aventis; and received support for attending meetings and/or travel from Sanofi-Aventis, Janssen-Cilag and has an unpaid leadership or fiduciary role at DGHO, DKG-Zertifizierungskommission and CCC Netzwerk. RB received payment or honoraria for lectures and participation in advisory boards for AbbVie, Amgen, AstraZeneca, Bayer, Bristol-Myers Squibb, Boehringer Ingelheim, Illumina, Janssen, Lilly, Merck Serono, Merck Sharp & Dohme, Novartis, Qiagen, Pfizer, Roche and Targos MP Inc.; and reports a leadership or fiduciary role as co-founder and co-owner of Gnothis Inc (SE) and Timer Therapeutics Inc (GE). JW received funding from Amgen, AstraZeneca, Bayer, Blueprint, Bristol-Myers Squibb, Boehringer Ingelheim, Chugai, Daiichi Sankyo, Janssen, Lilly, Loxo, Merck, Mirati, Merck Sharp & Dohme, Novartis, Nuvalent, Pfizer, Pierre-Fabre, Roche, Seattle Genetics, Takeda and Turning Point. The remaining authors declare no conflict of interest.
