# Supplementary material for: Evaluation of the effectiveness of a nationwide precision medicine program for patients with advanced non-small cell lung cancer in Germany: a historical cohort analysis
Source: Lancet Reg Health Eur. 2023 Nov 22;36:100788. doi: 10.1016/j.lanepe.2023.100788 (PMC10687333; doi:10.1016/j.lanepe.2023.100788)
Supplement: Supplementary Material, Figs. S1 and S2 and Table S1 [file mmc1.pdf]

Supplementary Material

Brief description of the national Network Genomic Medicine Lung Cancer (nNGM)

The national Network Genomic Medicine Lung Cancer (nNGM) was founded in 2018 and is funded by the German Cancer Aid. Until 2023, the nNGM included only patients with advanced (stage IIIB, IV) non-small cell lung cancer (NSCLC, all histologies). Since 2023, patients with early stage NSCLC are also included in the nNGM. Currently, 23 mostly academic centers provide harmonized and quality-controlled molecular multiplex testing, clinical recommendation, counseling during therapy, trial allocation and central evaluation of efficacy for around 400 regional nNGM network partners (private-practice based oncologists, community hospitals), which treat the majority of patients close to home. During the study period, 17 of such nNGM centers contributed data. All nNGM centers undergo a strict quality assurance program involving round robin tests for immunohistochemistry (IHC) and fluorescence in situ hybridization (FISH), conducted by the German Quality Initiative in Pathology (QuIP), as well as so-called performance tests (central retesting of routine diagnostic samples) and proficiency tests (diagnostic testing of centrally selected test samples) for next-generation sequencing (NGS)-based techniques organized by three reference nNGM centers (Cologne, Heidelberg, Munich). Molecular multiplex testing and clinical recommendation is fully covered by the majority of German statutory health insurances at initial diagnosis and in relapse. Molecular and clinical data are continuously collected in a central database. In 2022, the nNGM covered around 17,000 patients with advanced NSCLC, representing around two thirds of the target population, with an increasing tendency.

Supplementary Table 1. Inclusion and exclusion criteria

**Inclusion criteria**	**Codes**
Incident diagnosis lung cancer during 1st of April 2019 and 30th of June 2020	ICD-10: C34.0, C34.1, C34.2, C34.3, C34.8, C34.9
Health insured with AOK for at least two years prior to initial diagnosis	
Systemic oncologic therapy after initial diagnosis	ATC L01, OPS 8-54
**Exclusion criteria**	**Codes**
No main diagnosis lung cancer and lung cancer-related treatment (according to S3 guideline lung cancer[1]) within the first six months after initial diagnosis	
Lung resection after initial diagnosis	OPS 5-322, 5-323, 5-324, 5-325, 5-327, 5-328
Combination therapy of platinum-containing drugs and etoposide	ATC L01XA + L01CB01
Radiotherapy without metastases	OPS 8-52 + NOT (ICD-10: C77.0, C77.2, C77.3, C77.4, C77.5, C77.8, C78.-, C79.-)

Abbreviations: ICD-10 – International Classification of Disease, 10th revision; ATC – Anatomical Therapeutic Chemical code; OPS – operation and procedure code;

Reference: 1 AWMF. S3-Leitlinie Lungenkarzinom - Version 2.2. Leitlinienprogramm Onkologie. 2023; Available from: https://register.awmf.org/de/leitlinien/detail/020-007OL. Accessed: July 26, 2023.

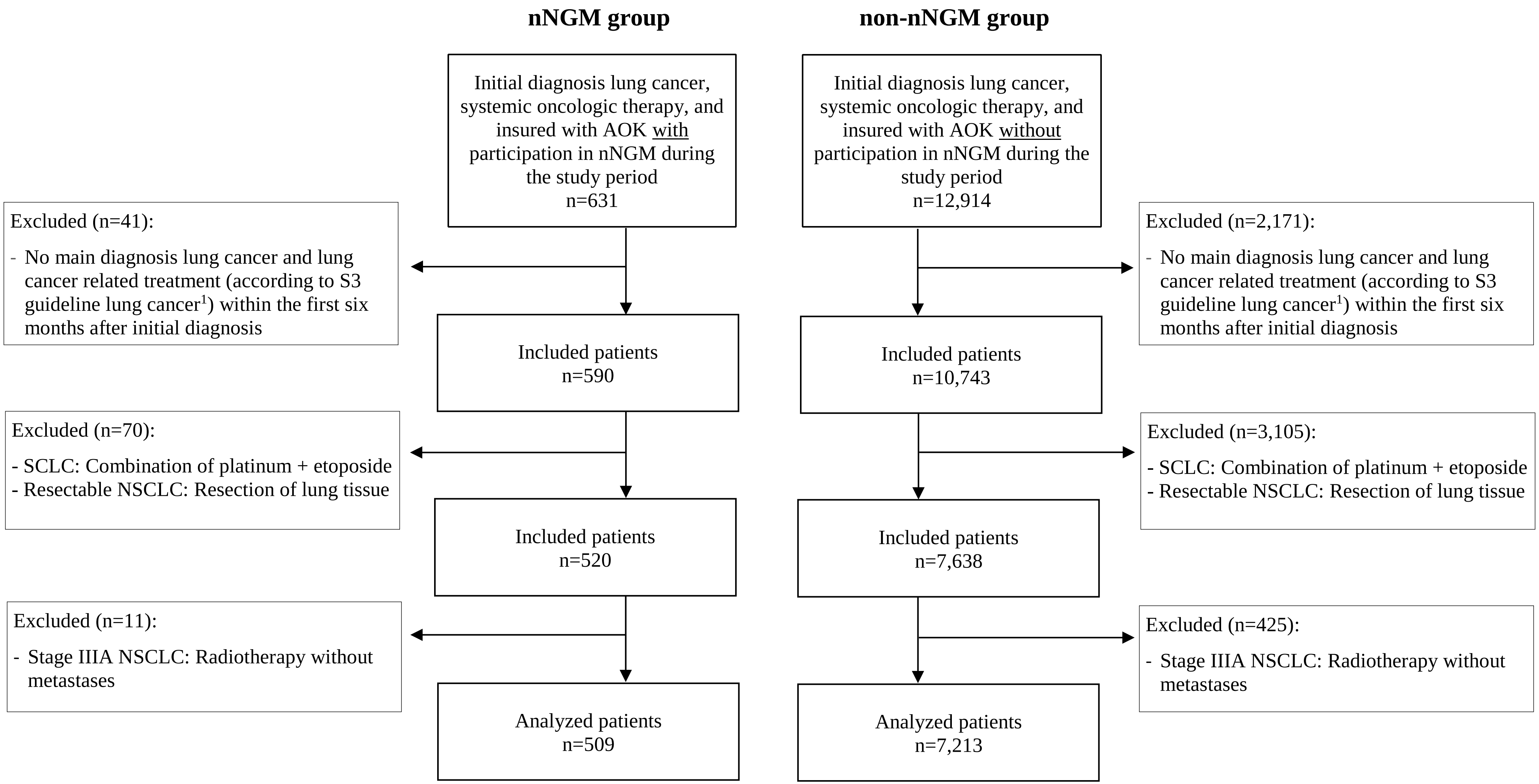

Supplementary Figure 1. Flow chart of patient selection for the nNGM group and non-nNGM group

Reference: 1. AWMF. S3-Leitlinie Lungenkarzinom - Version 2.2. Leitlinienprogramm Onkologie. 2023; Available from: https://register.awmf.org/de/leitlinien/detail/020-007OL. Accessed: July 26, 2023.

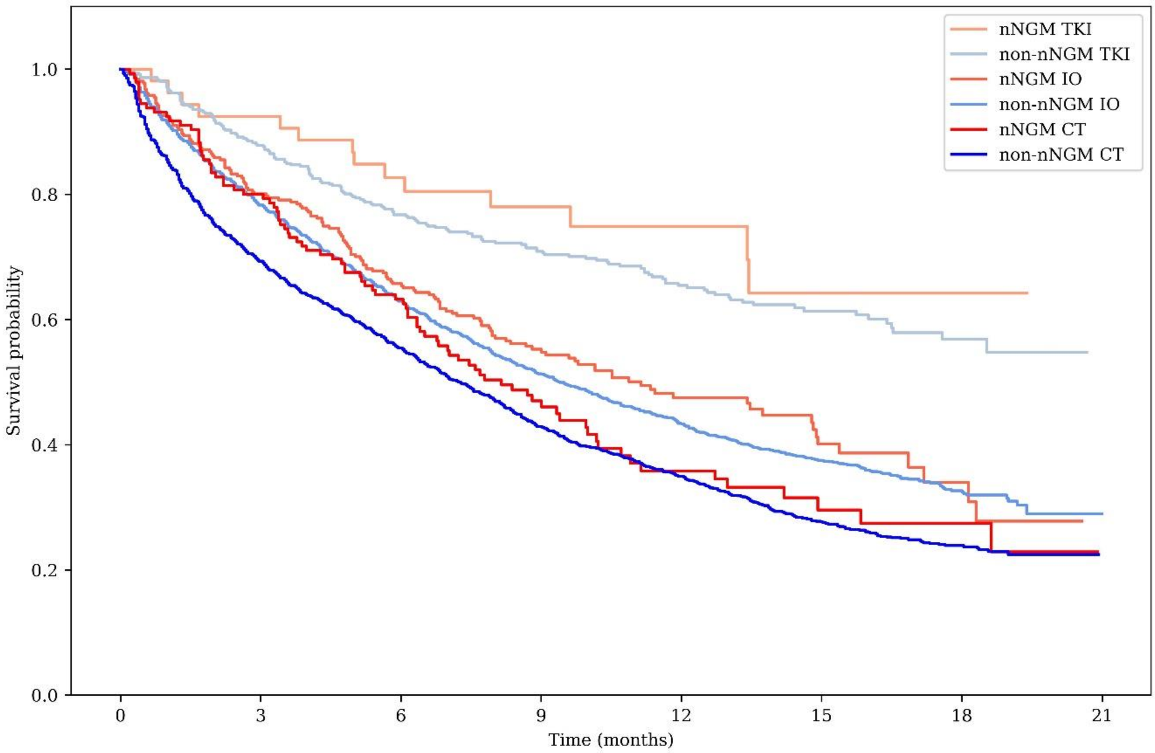

	0	3	6	9	12	15	18	21
**nNGM TKI**								
At risk	53	49	37	29	17	9	3	0
Censored	0	0	7	13	24	30	36	39
Events	0	4	9	11	12	14	14	14
**non-nNGM TKI**								
At risk	452	397	346	261	186	116	44	0
Censored	0	0	1	62	120	180	246	289
Events	0	55	105	129	146	156	162	163
**nNGM IO**								
At risk	311	249	188	119	67	33	12	0
Censored	0	0	17	59	97	124	142	152
Events	0	62	106	133	147	154	157	159
**non-nNGM IO**								
At risk	3883	3039	2436	1534	981	517	145	0
Censored	0	0	8	505	856	1208	1537	1674
Events	0	844	1439	1844	2046	2158	2201	2209
**nNGM CT**								
At risk	145	116	87	48	29	14	7	0
Censored	0	0	5	23	32	43	49	55
Events	0	29	53	74	84	88	89	90
**non-nNGM CT**								
At risk	2878	1994	1588	1048	685	375	127	0
Censored	0	1	8	212	404	591	799	921
Events	0	883	1282	1618	1789	1912	1952	1957

Supplementary Figure 2. Kaplan-Meier survival curve comparing overall survival since the start of first-line therapy between the nNGM group and the non-nNGM group, differentiated by treatment within the first six months after the start of therapy
